# A cross-lagged analysis of the relationship between short video overuse behavior and depression among college students

**DOI:** 10.3389/fpsyg.2024.1345076

**Published:** 2024-07-17

**Authors:** Dongning Zhang, Yifu Yang, Muzhen Guan

**Affiliations:** School of Public Health, Xi’an Medical University, Xi’an, China

**Keywords:** short video overuse behavior, depression, cross-lagged analysis, college students, gender differences

## Abstract

**Introduction:**

Watching short videos on mobile phones is currently a very prevalent phenomenon. It has been found in research that excessive use of short videos is closely related to depression. The aim of this study is to investigate the relationship between short video overuse behavior and depression among college students as well as the gender differences that are present in such relationship.

**Methods:**

A follow-up measurement was conducted on 331 college students using the Short Video Usage Behavior Scale and the Epidemic Research Center Depression Scale with an interval of 2 months.

**Results:**

(1) Correlation analysis revealed a significant positive correlation between short video overuse behavior and depression, whether measured at the same or different time points, repeated measures ANOVA indicates that short video overuse behavior and depression have strong stability within the interval between two measurements. (2) Pre-test short video overuse behavior could significantly and positively predict post-test depression, whereas pre-test depression could not significantly predict post-test short video overuse behavior. (3) The cross-lagged effect between short video overuse behavior and depression showed no gender differences.

**Discussion:**

These findings indicate that, for college students, short video overuse behavior may increase the risk of depression, whereas depression cannot induce short video overuse behavior.

## 1 Introduction

Numerous studies have shown that internet addiction and depression often occur simultaneously, and internet addiction can increase the risk of depression, making it an important predictor of depression (Oh et al., 2013; Ciarrochi et al., 2016). In recent years, with the development of mobile Internet technology and the emergence of smart phones, mobile phone addiction has become a new form of internet addiction. Some studies investigated the potential impact of mobile phone addiction on individual depression and found a significant positive correlation between the two (Jun, 2016; Alhassan et al., 2018; Yang et al., 2019). However, some studies have found that different ways of using mobile phones have different types of impact on mental health. For instance, recreational activities such as online gaming, watching TV, and listening to music appear to have a stronger impact on mental health, whereas online courses do not (Huang et al., 2023). Previous studies related to mobile phone addiction mainly focused on issues regarding social networking. However, in recent years, watching, publishing, and sharing short videos have become increasingly common ways of using mobile phones. Compared with traditional social media, short video applications not only have functions such as entertainment, social interaction, and information searching, but they also have the “personalized recommendation” function that can greatly reduce the cost of obtaining information, allowing users to enjoy a great deal of mental pleasure while also causing the problem of short videos overuse behavior. Short video overuse behavior (or short video addiction) refers to an individual’s inability to effectively control their short video usage behavior, which in turn has a negative impact on the individual, this behavior belongs to a branch of Internet overuse behavior (or internet addiction) (Zhang et al., 2019). An increasing number of studies have found that short videos’ traits of being easy to use and entertaining have facilitated people’s tendency to overuse, making it difficult for them to suppress their impulses. For example, approximately 22% of TikTok users spend more than 1 h per day on the application (Biznext, 2018). Studies have shown that short video overuse behavior can have negative effects, such as causing problems in interpersonal relationships (Huang et al., 2021; Li X. et al., 2021), inducing sleeping disorders (Hu et al., 2021), reducing subjective happiness (Ye et al., 2022), having a detrimental impact on psychological health (Zhang Q. et al., 2023). Research has also shown that short video overuse behavior can lead to depression (Liang et al., 2020). This effect appears to be especially strong in young adults who already have high levels of depression (Yao et al., 2022). The analysis of longitudinal cross-lagged panel network data shows that the “conflict” in Short Video Addiction and the “sad mood” in depression may serve as bridge symptoms linking the co-occurrence of these two mental health issues (Qu et al., 2024). However, there are other studies that did not find a robust relationship between watching short videos and psychopathological symptoms (Huang et al., 2023).

Research has shown a complex relationship between internet overuse behavior and depression (Morgan and Cotten, 2003; Yao and Zhong, 2014). Social displacement theory posits that internet overuse can reduce social activities between users and their family and friends while also replacing face-to-face communication with virtual communication, thereby decreasing one’s social support, sense of security, and sense of belonging, resulting in depressive symptoms (Kraut et al., 1998). Longitudinal studies have found that there is a bidirectional predictive effect between internet overuse behavior and depression (Xun et al., 2013; Tian et al., 2022), and those classified as heavy users of computer, social media, and mobile phone subsequently experience more severe long-term stress, depression, and sleep disorders (Thomée et al., 2011). However, the self-medicine theory (Negative reinforcement models) posits that using internet can regulate and alleviate emotions (Elhai et al., 2017), for example, depressed patients can use mobile phones as a way to deal with depression and negative emotions (Snodgrass et al., 2014; Kim et al., 2015). Therefore, in order to eliminate these psychological pressures and negative emotions, individuals will eventually develop excessive use of the Internet (Kim et al., 2017). The model of problematic internet use suggests that individuals facing pressure and experiencing negative emotions may seek comfort online to alleviate their pain (Kardefelt-Winther, 2014; Sun and Zhang, 2021). Although this method may temporarily alleviate emotional pain, it may lead to social isolation and dependence on the Internet and bring crisis in social support and social adaptability (Kardefelt-Winther, 2014).

As a new form of internet addiction, this study speculates that there is a similar bidirectional relationship between overuse of short videos and depression. On the one hand, overuse of short videos can interfere with an individual’s normal life, interpersonal communication, and lead to depression. On the other hand, individuals with negative emotions such as depression may also use short videos to comfort or compensate for their own pain. At present, no research has found the above relationship. In order to obtain more favorable evidence, this study decided to adopt a longitudinal cross lagged research method. A survey shows that college students are the main group currently using short videos, with a detection rate of 21.6% for their overuse of short videos (Li J. et al., 2021). College students are in the transitional stage from late adolescence to early adulthood, facing many changes and challenges in life, and are a high-risk group for depression (You et al., 2016). Therefore, short videos overuse behavior among college students will be regarded as the main research object of our study.

Research has found that there are more men than women with internet addiction (Morahan-Martin and Schumacher, 2000; Ko et al., 2005; Chen et al., 2007; Zhang et al., 2014; Tian et al., 2017), but the opposite phenomenon occurs in mobile phone addiction, with women significantly outnumbering men (Hong et al., 2012; Van Deursen et al., 2015). Mental health problems caused by problematic internet use are moderated by gender factors (Frison and Eggermont, 2016), for example women often spend more time online chatting and participate in social networking activities, which can easily lead to a decrease in social support (Liang et al., 2016), women are more likely than men to experience upward social comparison when using social networking sites, which can lead to negative emotions such as jealousy (Nesi and Prinstein, 2015). Other studies have proposed that watching short videos has a stronger effect on women than men in certain aspects, such as sleeping and physical satisfaction (Liang et al., 2020; Hu et al., 2021). Therefore, we speculate that the relationship between short video overuse behavior and depression is closer among women than among men.

Considering the abovementioned theories and studies, we posit that bidirectional effects may be present in the relationship between short video overuse behavior and depression among college students. Thus, we propose the following hypotheses:

Hypothesis 1: Short video overuse behavior that occurs at an earlier stage can positively predict later depression.

Hypothesis 2: Depression during an earlier stage can positively predict later short video overuse behavior.

Hypothesis 3: The relationship between short video overuse behavior and depression differs by gender.

## 2 Materials and methods

### 2.1 Sample size estimate

We calculated the sample size for this study according to formula *N* = *Z*^2^_1-α/2_*P* (1−*P*)/*E*^2^. *N* is the sample size; *Z*_1-α/2_ is the area under standard normal distribution according to α = 0.05; *Z*_1-α/2_ takes a value of 1.96; and *E* is the allowable error, set at 5%, and *P* represents the depression detection rate, According to literature, the detection rate of depression in CES-D is approximately 23.1% (Chen et al., 2022). The final calculated sample size is 273 cases. Based on a 20% invalid questionnaire ratio, the survey subjects should include at least 330 people.

### 2.2 Participants

Some studies suggest that a shorter time interval between two waves of measurement may produce the bigger effect size. Therefore, this study sets 2 months as the optimal lag time (Dormann and Griffin, 2015). Participants were selected using convenience sampling with students in different grades at three universities in Xi’an. The first survey was conducted at the end of March 2023 (T1), from which 435 valid participants were obtained, including 194 males and 241 females. The second survey was conducted at the beginning of June 2023 (T2), from which 408 valid participants were obtained, including 185 males and 223 females. After removing all the invalid responses, 331 questionnaires were collected with matching responses from both T1 and T2, turnover rate is 23.91%. The measurements were administered centrally in class units, with trained counselors serving as the main examiners. The purpose and requirements were explained in detail to all participants before the survey. The questionnaires were completed anonymously (each participant was randomly assigned a number, which remained the same for both measurements), and the survey time was limited to 10 min. The Medical Ethics Review Committee of Xi’an Medical College approved this study, and all participants signed informed consent forms.

### 2.3 Procedures

The measurements were administered centrally in class units, with trained counselors serving as the main examiners. The purpose and requirements were explained in detail to all participants before the survey. The questionnaires were completed anonymously (each participant was randomly assigned a number, which remained the same for both measurements), and the survey time was limited to 10 min. The Medical Ethics Review Committee of Xi’an Medical College approved this study, and all participants signed informed consent forms.

### 2.4 Measures

#### 2.4.1 Short video usage behavior scale

Wang J. et al. (2020) compiled the Short Video Usage Behavior Scale, from which our study used the “overuse” dimension. This dimension has seven total items: “watching short videos on mobile phones takes up a lot of my time”; “generally, I watch short videos on my phone when I am free”; “I frequently turn on my phone to watch the latest short videos”; “my leisure and entertainment in my life mainly consist of watching short videos on my phone”; “I try to reduce my time spent watching short videos on my phone, but without success”; “because of watching short mobile phone videos, my interaction with family and friends has decreased”; and “because of watching short mobile phone videos, my sleep has decreased.” The participants responded using a five-point scale (1 = “completely inconsistent” and 5 = “completely consistent”). The higher the score, the more serious the short video overuse behavior. In this study, the retest reliability is 0.64, with *p* < 0.01. The scale demonstrated a Cronbach’s α coefficient of 0.92 at T1 and 0.90 at T2.

#### 2.4.2 Center for epidemiologic studies depression scale

The Center for epidemiologic studies depression scale (CES-D), compiled by Radloff (1977) and reformulate by Zhang et al. (2010), was used in this study. Participants fill out a questionnaire based on the symptoms and frequency they have experienced in the past week on a scale of 0–3. The scale content includes four factors: depressed affect, positive affect, somatic and retarded activity, and interpersonal. The respective items are as follows: “I think even with the help of friends, I cannot get rid of this kind of frustration”; “I think there is hope for the future”; “I do not want to eat anything; I have a bad appetite”; and “I feel that others dislike me.” The higher the total score, the more severe the depression. In this study, the retest reliability is 0.63, with *p* < 0.01. Cronbach’s *α* was 0.92 and 0.91 at T1 and T2, respectively.

### 2.5 Data analysis

SPSS25.0 was used for descriptive statistics and correlation analysis, as well as calculation of test–retest reliability, common method bias test and internal consistency reliability analysis. Repeated measures ANOVA was then used to test the average changes in short video overuse behavior and depression in two measurements. Longitudinal invariance analysis, cross-lagged analysis, and multi-group gender analysis were all conducted using Mplus 7.0. The model estimation method adopts robust maximum likelihood estimation (MLR). The model fitting index adopts χ^2^/df, CFI, TLI, and RMSEA. Chi square test is used in model comparison in multi-group analysis.

## 3 Results

### 3.1 Common method bias test

Using Harman single-factor test to determine if there is a common method bias. Include all 54 test results from the two measurements of short video overuse behavior and depression in the analysis. The results displayed that five and four factors had eigenvalues greater than 1 in the two measurements, respectively. The first factor explained 31.12 and 33.51% of the variance at T1 and T2, respectively. Both values were less than 40%, indicating no obvious common method bias.

### 3.2 Correlation analysis and difference tests for short video overuse behavior and depression

The final sample included 152 male students (45.92% of the total sample) and 179 female students (54.08%), with a mean age of 20.46 ± 1.24 years. The results of chi-square test and independent sample *t*-test indicate that, there were no significant differences in gender (χ^2^ = 0.84, *p* > 0.05), age (*t* = 1.23, *p* > 0.05), and pre-test short video overuse behavior (*t* = −0.95, *p* > 0.05) and depression (*t* = −0.95, *p* > 0.05) between the valid and missing samples. This indicates that the loss of research participants in this study is a non-structural loss. Table 1 shows that there is a significant positive correlation between short video overuse behavior and various dimensions of depression in both tests. The correlation coefficient between the two short video overuse behaviors is 0.64, the correlation coefficient between short video overuse behavior and various dimensions of depression is between 0.15 and 0.31, and the correlation coefficient between various dimensions of depression is between 0.22 and 0.78.

**Table 1:** Descriptive and correlation analyses of short video overuse behavior and depression in the pre- and post-tests (*n* = 331).

	M ± SD	1	2	3	4	5	6	7	8	9
1. SV_T1_	19.42 ± 6.07	1								
2. DA_T1_	4.05 ± 4.51	0.26^**^	1							
3. PA_T1_	4.68 ± 3.36	0.15^**^	0.46^**^	1						
4. SA_T1_	3.93 ± 3.36	0.28^**^	0.77^**^	0.36^**^	1					
5. IP_T1_	0.81 ± 1.08	0.18^**^	0.78^**^	0.37^**^	0.59^**^	1				
6. SV_T2_	19.84 ± 5.65	0.64^**^	0.21^**^	0.15^**^	0.24^**^	0.17^**^	1			
7. DA_T2_	4.14 ± 3.99	0.31^**^	0.63^**^	0.33^**^	0.53^**^	0.49^**^	0.31^**^	1		
8. PA_T2_	4.57 ± 3.17	0.18^**^	0.38^**^	0.40^**^	0.35^**^	0.31^**^	0.17^**^	0.49^**^	1	
9. SA_T2_	4.29 ± 3.22	0.26^**^	0.49^**^	0.32^**^	0.55^**^	0.35^**^	0.32^**^	0.78^**^	0.45^**^	1
10. IP_T2_	0.95 ± 1.09	0.26^**^	0.45^**^	0.23^**^	0.37^**^	0.33^**^	0.22^**^	0.74^**^	0.42^**^	0.64^**^

M, Mean; SD, Standard deviation; SV, Short video overuse behavior; DA, Depressed affect; PA, Positive affect; SA, Somatic and retarded activity; IP, Interpersonal; T1, The pre-test; T2, The post-test; ^*^p < 0.05, ^**^p < 0.01, ^***^p < 0.001; also applicable to the other forms shown below.

Table 2 shows the mean and standard deviation of pre- and post-test short video overuse behavior and depression among male and female students. Using measurement time (including pre-test T1 and post-test T2) as the within-subjects variable, gender as the between-subjects variable, and depression as the dependent variable, a 2 × 2 repeated-measures ANOVA was conducted. The results demonstrated that the main effect of measurement time was not significant [*F*(1, 329) = 0.75, *p* > 0.05]. There is a significant gender main effect [*F*(1, 329) = 9.33, *p* < 0.01, η^2^_p_ = 0.03], with men experiencing significantly higher levels of depression than women. The interaction between measurement time and gender was significant [*F*(1, 329) = 3.90, *p* < 0.05, η^2^_p_ = 0.01]. Taking measurement time (including pre-test T1 and post-test T2) as the within-subjects variable, gender as the between-subjects variable, and short video overuse behavior as the dependent variable, a 2 × 2 repeated-measures ANOVA was performed. The results showed that the main effect of measurement time [*F*(1, 329) = 2.07, *p* > 0.05], main effect of gender [*F*(1, 329) = 1.67, *p* > 0.05], and the measurement time and the interaction effect of gender [*F*(1, 329) = 2.21, *p* > 0.05] were not significant.

**Table 2:** Mean and standard deviation of short video overuse behavior and depression in men and women pre- and post-tests (*n* = 331).

	Men (M ± SD)	Women (M ± SD)	t
SV_T1_	20.05 ± 6.66	18.88 ± 5.49	1.715
SV_T2_	20.03 ± 6.14	19.68 ± 5.21	0.555
Depression_T1_	15.57 ± 11.41	11.67 ± 8.65	3.46^**^
Depression_T2_	15.05 ± 10.99	12.99 ± 8.28	1.897

^**^p < 0.01.

### 3.3 Cross-lagged analysis of short video overuse behavior and depression

Before conducting cross-lagged analysis, the longitudinal invariance of short video overuse behavior and the CES-D in two measurements were first tested. Configural invariance, metric invariance, and scalar invariance models were established for the two measurements of short video overuse behavior and depression, respectively. The model fitting results are shown in Table 3. According to the chi-square test results, the model differences are not significant; therefore, cross-lag analysis can be performed.

**Table 3:** Longitudinal invariance test of the short video overuse behavior scale and the center for epidemiologic studies depression scale.

	Model	RMSEA	CFI	TLI	*χ* ^2^	df	Δ*χ*^2^	Δ*df*	*p*-value
SV	Unconstrained	0.070	0.971	0.957	161.563	62			
	Metric	0.067	0.972	0.961	162.956	66	1.393	4	0.845
	Scalar	0.065	0.970	0.962	176.194	73	13.238	7	0.067
Depression	Unconstrained	0.034	0.963	0.957	752.347	542			
	Metric	0.033	0.964	0.96	760.349	557	8.002	15	0.923
	Scalar	0.033	0.964	0.96	776.601	571	16.252	14	0.298

**Figure 1:**
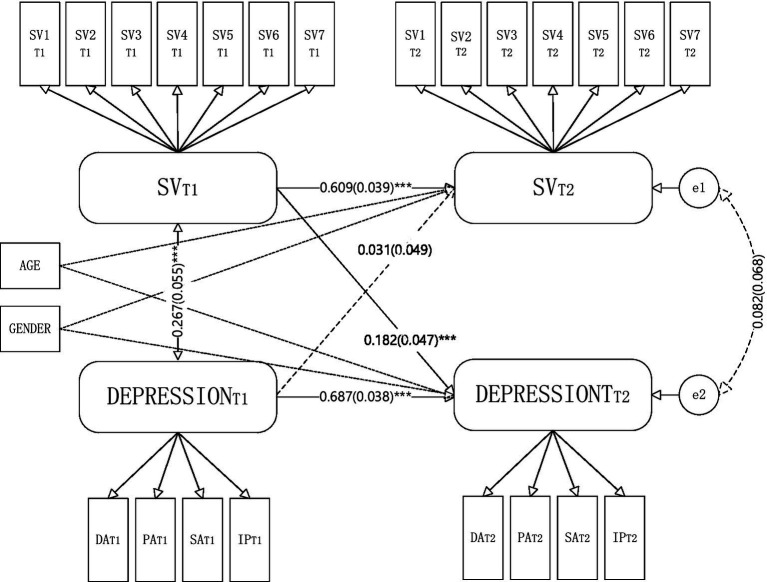
Cross-lagged analysis of short video overuse behavior and depression. T1, The first survey; T2, The second survey; ^***^p < 0.001. Standardized coefficients and bootstrapped standard errors (in parentheses) are presented. The path coefficients marked by the solid line are all significant standardized path coefficients, while the dashed line represents insignificant paths.

Based on theoretical assumptions and after controlling for age and gender factors, we established a cross-lagged model between short video overuse behavior and depression. The maximum likelihood estimation method test showed that the various indicators of the model fit well: χ^2^/df = 2.225, *p* < 0.001, CFI = 0.944, TLI = 0.934, and RMSEA = 0.061. As Figure 1 shows, the autoregressive pathways from T1 short video overuse behavior to T2 short video overuse behavior (β = 0.609, SE = 0.039, *p* < 0.001) and from T1 depression to T2 depression (β = 0.687, SE = 0.038, *p* < 0.001) were significant. The cross-lagged path test results found that T1 short video overuse behavior significantly and positively predicted T2 depression (β = 0.182, SE = 0.047, *p* < 0.001); however, T1 depression did not significantly predict T2 short video overuse behavior (β = 0.031, SE = 0.049, *p* > 0.05).

Using multiple sets of structural equation models, we tested whether the relationship pattern between variables shown in Figure 1 has gender differences. First, a baseline model with free estimation of all paths in the men and women group models (M1) was found to have a good fit (χ^2^/df = 1.890, CFI = 0.932, TLI = 0.920, and RMSEA = 0.052). Second, the measurement weights model (M2), which limits the weights of corresponding indicators in the latent variables to make them equal between the men and women models was also found to have a good fit (χ^2^/df = 1.914, CFI = 0.927, TLI = 0.918, and RMSEA = 0.053). Finally, a structural weights model (M3) established on the basis of model M2, with equal autoregressive paths and cross lagged paths in both the men and women models, was found to have a good fit (χ^2^/df = 1.865, CFI = 0.926, TLI = 0.922, and RMSEA = 0.051). The chi-square difference test was used to test the M2 and M1 models, and the results showed that Δχ^2^(18) = 43.652, *p* < 0.05, ΔCFI = −0.005, ΔTLI = −0.002, and ΔRMSEA = 0.001. Therefore, based on comprehensive judgment, there is no significant difference between the two models. The test results of M3 and M2 show that Δχ^2^(26) = 28.608, *p* > 0.05, the difference between the two models is equally insignificant.

## 4 Discussion

### 4.1 Correlation analysis and repeated-measures ANOVA

In this study, correlation analysis revealed a significant positive correlation between short video overuse behavior and depression, whether measured at the same or different time points. This result validates previous research (Liang et al., 2020; Zhu et al., 2024), meanwhile, it indicates that during the time interval between two measurements, the more the short videos overuse behavior, the more severe the depression, and vice versa. Repeated-measures ANOVA indicated that college students’ short video overuse behavior and depression showed strong stability during the interval between measurements and did not easily change over time. Regarding depression, the results of this study showed that T1 depression was significantly higher in men than in women. Although numerous studies have shown that women are more prone to depression than men, multiple meta-analyses have shown that male college students in China either have more severe depression than female students or are not any different from female students in terms of susceptibility to depression (Tang et al., 2013; Wang M. et al., 2020; Zhang et al., 2020). In addition, the results of a cross temporal meta-analysis from 2000 to 2017 showed that male (vs. female) college students’ level of depression increased more rapidly (Feng et al., 2020). Compared with women in other social groups, female college students do not have differences in social status, economic burden, and family responsibility compared to male students. This may be the reason why female college students are not necessarily more likely to develop depression than male students. For the short video overuse behavior, this study did not find any gender differences, which is inconsistent with the results of previous research on internet addiction and mobile phone addiction. The results regarding internet addiction suggest that more men than women are addicted (Chou and Hsiao, 2000; Morahan-Martin and Schumacher, 2000), while the results regarding mobile phone addiction suggest that more women than men are addicted (Hong et al., 2012; Van Deursen et al., 2015). This study suggests that this may be due to the rich information and personalized recommendation function of short video platforms, allowing both men and women to obtain their favorite information on the platforms, thus there is no gender difference.

### 4.2 Cross-lagged analysis

The cross-lagged analysis showed that T1 short video overuse behavior could significantly and positively predict T2 depression; however, T1 depression could not significantly predict T2 short video overuse behavior. This indicates a potential causal relationship between short video overuse behavior and depression among college students. Specifically, short video overuse behavior may induce or aggravate depression; however, depression will not cause short video overuse behavior. This finding supports Hypothesis 1 but does not support Hypothesis 2.

These findings support social displacement theory. Owing to the strong appeal and communication power of short videos on mobile phones, some students with insufficient self-control become addicted to them, which reduces real-life interpersonal interactions, interferes with normal learning activities and sleep, reduces social support, and decreases subjective well-being, resulting in depressive symptoms. Research on the use behavior of social networking sites (SNS) has found that passive use on social network sites can lead to individuals experiencing upward social comparison, and the social rank theory of depression suggests that upward social comparison is a key factor in inducing individual depression (Sloman et al., 2003; Burnette et al., 2017). Further, regarding short video platforms as a form of social network sites, previous surveys have shown that college students’ short video usage behavior is dominated by “passive viewing” (Li, 2020), which lacks interpersonal interaction and communication. Thus, viewers are easily misled by ostentatious and decorative information in videos, leading to upward social comparison, which in turn leads to depression.

This study found that depression does not predict subsequent short video overuse behavior. This result does not support the self-medical theory. Previous studies have found a bidirectional relationship between internet overuse behavior and depression. Short video social media platforms are a new form of social media that has emerged in recent years. Compared with traditional online gaming and social networking services, short video applications have personalized recommendation functions, that is, short video platforms will recommend information that matches the user’s personal preferences based on their characteristics. Secondly, the duration of short video information is extremely short, often ranging from a few seconds to a few minutes. Furthermore, complete video information has a stronger impact on users than text information. In addition, short video applications also have very simple video editing and uploading functions. The motivation and purpose of short video usage behavior may differ from traditional online applications or social platforms. The core element of traditional social media is social interaction, while the core element of short-form video apps is entertainment (Smith and Short, 2022; Chao et al., 2023). Therefore, research results from other social media or applications may not be applicable to short video applications (Zhang N. et al., 2023). In addition, this study used a 2-month time interval, which may also be the reason why depression does not affect short video overuse behavior. Research has shown that the relationship between depression and internet addiction is in a dynamic development, and the relationship between the two may vary at different measurement time points.

The multi-group analysis showed no gender difference in the relationship between short video overuse behavior and depression, this result is completely consistent with previous studies (Zhu et al., 2024). This result does not support hypothesis H3. Although studies on internet addiction and social networking have shown that women are more susceptible than men to depression caused by internet use behavior, this study did not find any unique effects of short video overuse behavior on women. This result may be related to the characteristics of short video use behavior itself, or to the relatively short 2 months time interval used in this study, it may fail to capture this difference.

In this study, we adopted a longitudinal cross-lagged design for the investigation of the relationship between short video overuse behavior and depression among college students. Aside from verifying the causal relationship between the two aforementioned variables on a theoretical level while enriching the content within the research area pertaining to the influence that short video overuse behavior has on the psychological health of short video users, the results of our study have also provided theoretical support for the social displacement theory. As for the meaning of our study from a practical standpoint, taking the prevalence of the behavior of using short video applications among current college students into consideration, policy makers and teaching staff of colleges should pay special attention on the potential negative impact that short video overuse behavior has on the psychological health of their students while making effort to guide them to use short videos in a more moderate manner, so that disturbances on studying and socialization, which might lead to depression, can be avoided.

### 4.3 Research limitations and prospects

Several limitations of this study along with directions for future research need to be noted. Firstly, this study used the survey approach for generalization purposes, the design fully relying on self-report data; therefore, the self-rated nature of the scales in our research may make answers biased. Secondly although this study used a cross-lagged design, which is more powerful than cross-sectional studies, to confirm the relationship between short video overuse behavior and depression, measurements were taken at only two time points, preventing the capture of dynamic changes in each variable. In addition, the time interval between the two measurements was relatively short. Therefore, future studies should extend the tracking duration and increase the number of measurements. Thirdly, only 331 participants participated in this study, which may be the reason why the research hypothesis does not fully match the final conclusion. In the future, the sample size should be further expanded to confirm the relationship between the two variables.

## 5 Conclusion

The current study demonstrates that both short video overuse behavior and depression among college students show strong stability and are not easily changed over time. Cross-lagged analysis shows that pre-test short video overuse behavior significantly and positively predict post-test depression, whereas pre-test depression does not significantly predict post-test short video overuse behavior. Further, no significant gender differences exist in the relationship between the two. This study suggests that short video overuse behavior may be a risk factor for depression in college students, and eliminating short video overuse behavior may reduce the likelihood of depression occurrence.

## Data availability statement

The raw data supporting the conclusions of this article will be made available by the authors, without undue reservation.

## Ethics statement

The studies involving humans were approved by Committee of Xi’an Medical College. The studies were conducted in accordance with the local legislation and institutional requirements. The participants provided their written informed consent to participate in this study.

## Author contributions

DZ: Conceptualization, Funding acquisition, Investigation, Methodology, Writing – original draft, Writing – review & editing. YY: Data curation, Investigation, Writing – original draft, Writing – review & editing. MG: Conceptualization, Investigation, Methodology, Writing – original draft.
